# An Algorithm for Automatic Osteotomy Plate Placement Planning in 3D: Application in Distal Radius Malunion

**DOI:** 10.1007/s11548-026-03576-5

**Published:** 2026-01-27

**Authors:** Eva van de Nes, Camiel J. Smees, Judith olde Heuvel, Anne J. H. Vochteloo, Gabriëlle J. M. Tuijthof

**Affiliations:** 1 Centre for Orthopaedic Surgery OCON, Hengelo, Overijssel the Netherlands; 2 Department of Biomechanical Engineering, University of Twente, Enschede, the Netherlands

**Keywords:** Distal radius malunion (DRM), Osteotomy planning, 3D-assisted surgery, Volar plate positioning, Automatic plate placement, Computer-aided surgery

## Abstract

**Purpose:**

Positioning of an osteosynthesis plate is a key step in the preoperative 3D planning processes for the design of patient-specific guides. This step requires considerable time and expertise. To increase 3D planning efficiency, this study aims to develop an automated plate positioning algorithm.

**Methods:**

A robust algorithm was developed to optimize osteosynthesis plate positioning on the distal radius, using STL properties and anatomical landmarks. The algorithm involved alignment, landmark detection, initial placement, and final optimization. Retrospective data of 34 planned radii and corresponding plate positions, including decimated and refined mesh versions, were used to compare algorithm output to manual placement based on runtime, Hausdorff distance, translation, and rotation (mean ± SD, 95% CI), thereby assessing robustness across different mesh resolutions.

**Results:**

The average run time for the algorithm was 18.3 ± 16.8 s (95% CI 12.4–24.1 s) compared to a manual placement time of 12.45 ± 4.56 min (single expert, *n* = 10, 95% CI 9.22–16.28 min). The mean unpaired maximum Hausdorff distance between manual and algorithm placements was 5.5 ± 2.5 mm (95% CI 4.6–6.4 mm). The mean rotation and translation differences were 4.9 ± 3.2° (95% CI 3.8–6.0°) and 3.3 ± 1.7 mm (95% CI 2.8–3.9 mm), respectively.

**Conclusion:**

In conclusion, while some manual adjustment remains necessary, the algorithm aids in reducing planning time and offers a modular, generalizable framework adaptable to other osteotomy-plate procedures, supporting clinical 3D planning.

## Introduction

CT-based 3D surgical planning is increasingly used in orthopedic surgery, enabling the creation of patient-specific guides (PSGs) that can reduce operative time and radiation exposure, and increases surgical precision and reproducibility [1].

A main limitation of PSGs is that 3D preoperative planning remains largely manual and time-consuming [2]. Reported planning times vary between 2 and 4 h depending on osteotomy type (excluding segmentation) [3], to 6 h at our institution for distal radius malunion planning, including segmentation, osteotomy definition, plate positioning, and PSG design.

Automation of the planning could reduce time and increase objectivity. A few studies, such as Carrillo et al. [4], have incorporated automated steps for plate-to-bone distance minimization, but these pipelines did not report plate positioning times or compare algorithmic output with manual planning. This study aims to develop a robust plate placement algorithm for distal radius osteotomies that (1) substantially reduces planning time through automation and (2) increases robustness by introducing objective and reproducible plate to bone alignment. The modular design of the algorithm is intended to allow future alteration and extension to other long bone osteotomies.

## Methods

**Fig. 1:**
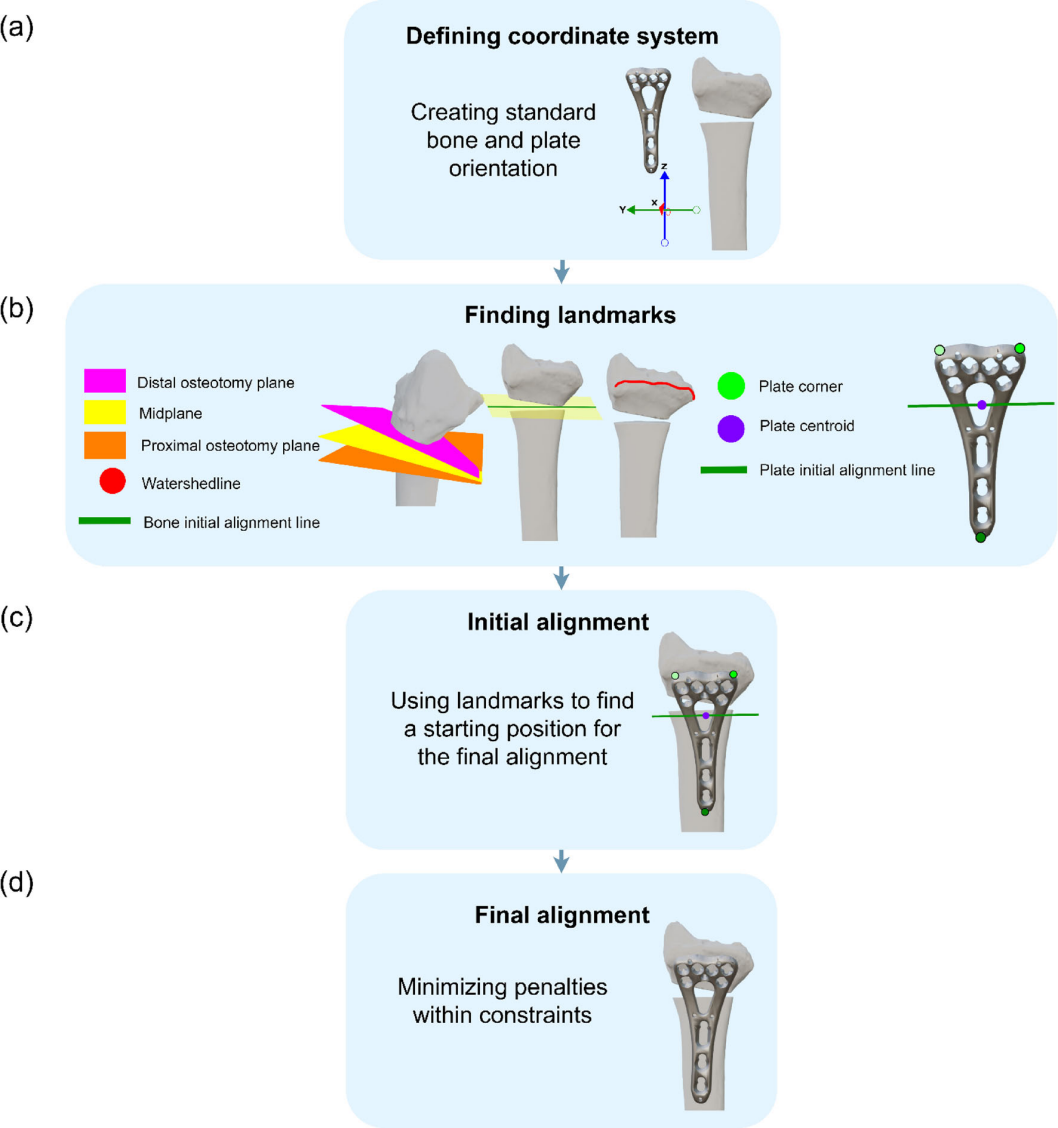
An algorithm overview, **a** Defining coordinate system, **b** Finding landmarks, **c** Initial alignment, **d** Final alignment

An automated algorithm was developed in Python 3.12 to facilitate automatic plate positioning (Fig. 1). Given the broad scope and evaluation challenges across bone types, the initial focus was limited to the distal radius for model development and validation. The algorithm consists of four main steps: (a) Defining a coordinate system, establishing standard bone and plate orientation; (b) finding landmarks, identifying key landmarks on the bone and plate for initial positioning; (c) initial alignment by roughly positioning the plate, and (d) final alignment by optimizing the position using penalties and constraints.

### Algorithm Overview

#### Input and Output

**Fig. 2:**
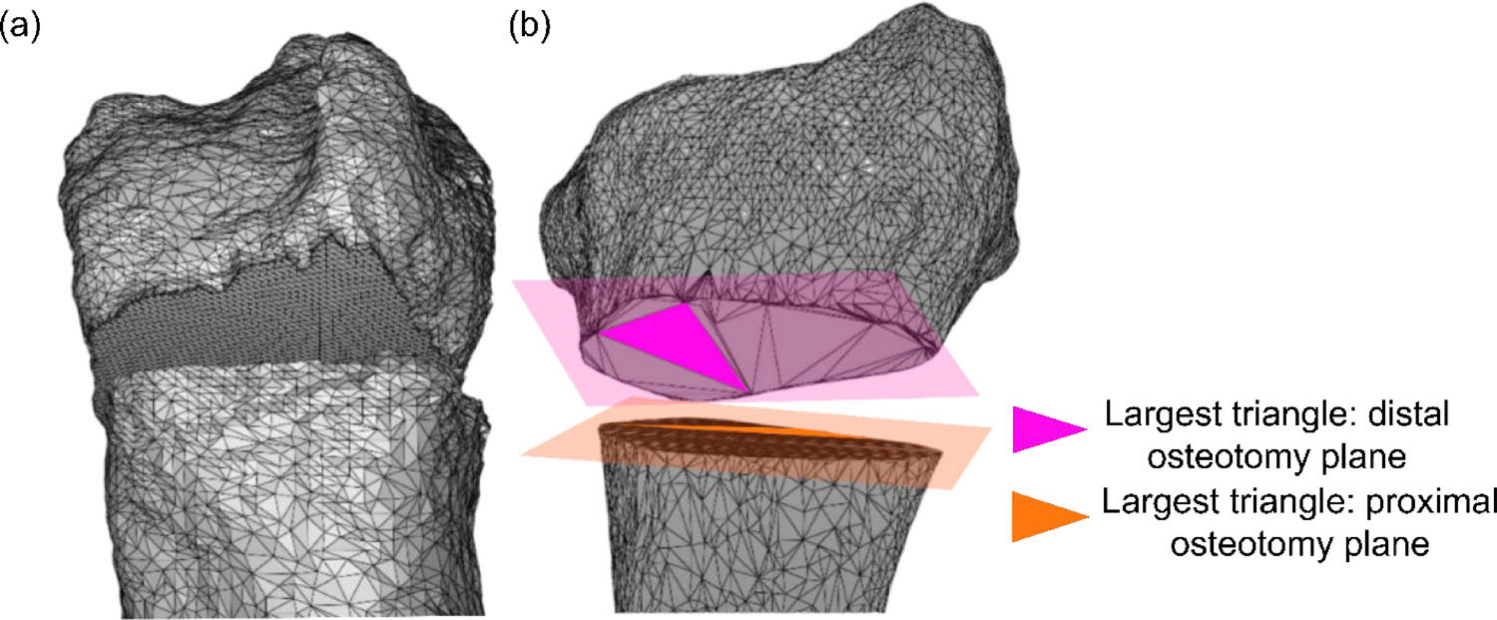
Visualization of STL files required for the algorithm **a** A non-optimized radius STL mesh, the flat surfaces consist of equally sized triangles. **b** A properly decimated radius STL mesh, with the distal osteotomy plane (pink) and proximal osteotomy plane (orange)

The segmented 3D bones consist of a combination of the distal radius with assigned osteotomy plane and the remainder of the radius with assigned osteotomy plane (Fig. 2). Subsequently, the bone model is preprocessed by mesh decimation [5], preparing the triangle sizes on the planar surfaces to be used in the algorithm (Fig. 2). The bone model is used as input to the algorithm including other parameters (Table 1). The distal and proximal osteotomy planes, identified by their largest mesh triangles, are used in Steps a and b to achieve alignment (Fig. 1ab).

**Table 1:** Overview of algorithm input and output

Inputs	- Optimally meshed STL Bone file (Fig. 2b) - Plate STL file - User indication of bone and plate type (“left” or “right”)
Outputs	- Bone STL aligned to standardized orientation - Plate STL positioned on the bone closely to desired clinical position

#### Algorithm Step (a): Defining Coordinate System

**Fig. 3:**
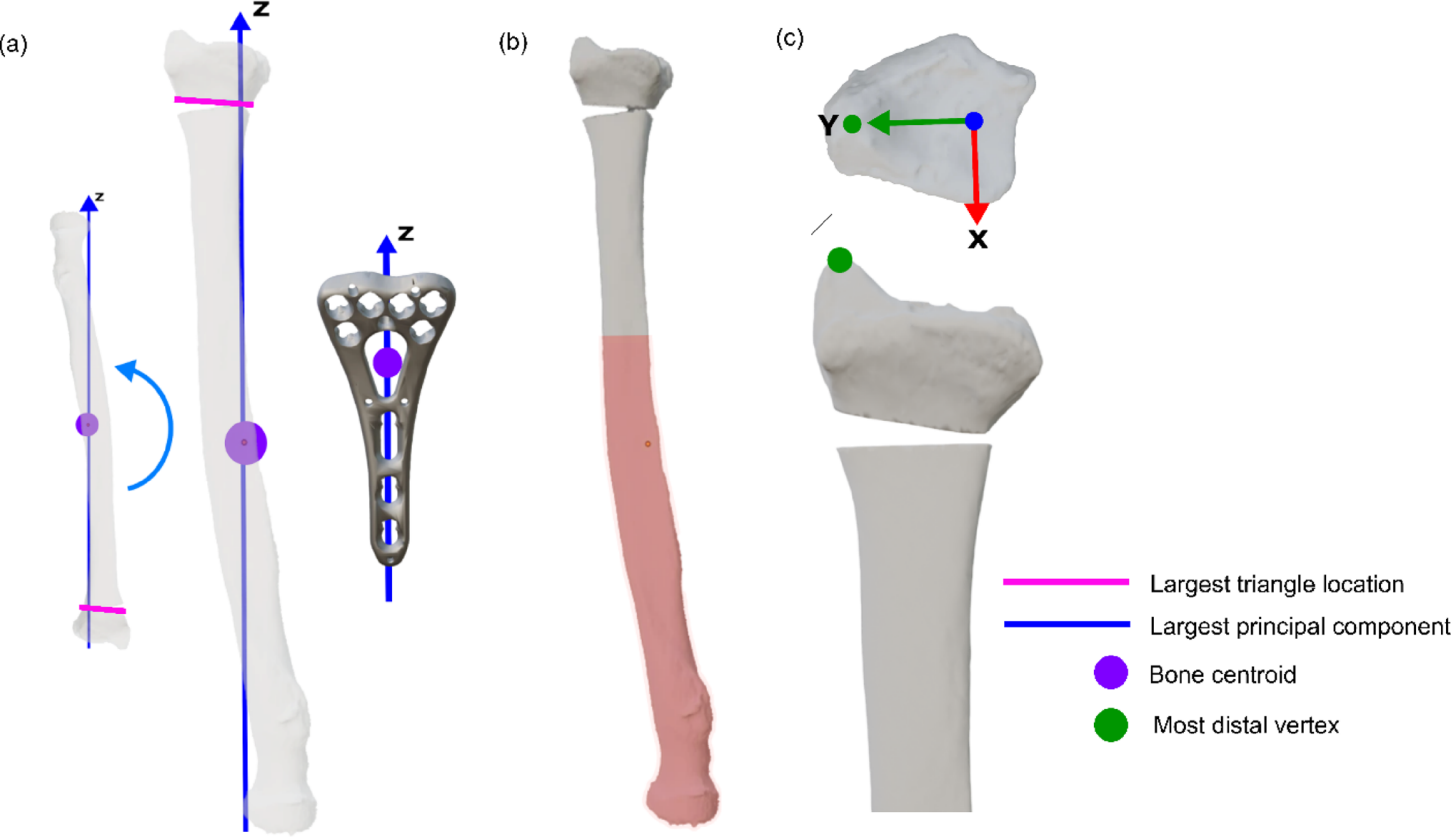
Algorithm step a in detail: defining coordinate system. **a** Defining the Z-axis (dark blue line) with PCA, ensuring the largest triangle (pink line) has a bigger Z-coordinate than the radius centroid (purple circle), aligning the plate’s largest principal component with this same axis. **b** Creating a new mesh from the upper portion of the bone mesh. **c** Defining the positive y-axis using the most distal vertex (green circle). The x-axis points toward the volar radius

The origin of the coordinate system is defined as the bone mesh centroid. Principal component analysis (PCA) is applied separately to the radius and plate, aligning their longest principal axes with the Z-axis. The radius is considered upright when its distal part points in the positive Z-direction, verified by checking whether the distal and proximal osteotomy planes have a larger Z-value than the radius mesh centroid (Fig. 3a). If not, the mesh is flipped in the Z-direction.

The distal bone segment is isolated by retaining only vertices with Z-values above an adjustable threshold set to 40 mm (Fig. 3b), which corresponds to 17% of the average radius length [6]. This value ensures the distal segment is large enough to encompass all clinically relevant plate positions, while reducing mesh size and thus computational load. The positive Y-axis is defined as perpendicular to the Z-axis and directed through the biggest Z-coordinate (i.e., the styloid process, Fig. 3c). The X-axis is set orthogonally pointing toward the radius’s volar aspect.

#### Algorithm Step (b): Finding Landmarks

With the bone oriented, four anatomical landmarks are defined to guide reproducible initial plate positioning: the distal and proximal osteotomy planes, the midplane, and the watershed line.

**Fig. 4:**
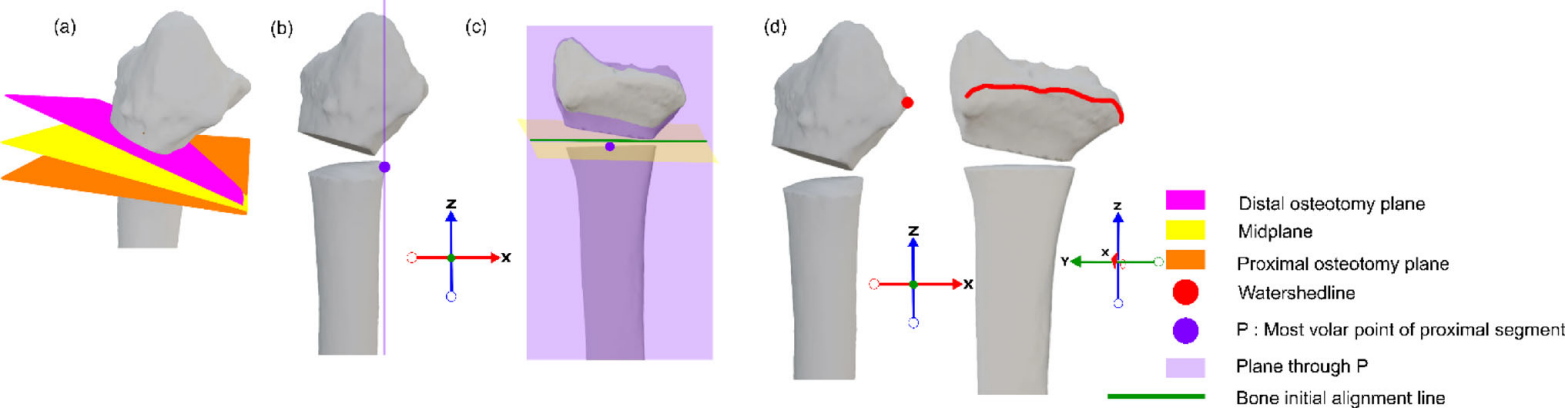
Algorithm step b in detail: Finding landmarks; bone. **a** The distal (pink) and proximal (orange) osteotomy planes are defined based on the largest triangles, and halfway from them, the midplane (yellow). **b** Point P, the most volar point on the proximal segment, through which a plane (purple) is constructed, parallel to the Y–Z plane. **c** The initial bone alignment line (green) is the intersection between the midplane and the plane through point P. **d** The watershed line (red) is identified as the most volar (highest X-coordinate) line on the distal bone segment

The midplane is identified as the average of the osteotomy planes (Fig. 4a). Next, the most volar point on the proximal segment is used to construct a plane parallel to the YZ-plane (Fig. 4b); its intersection with the midplane defines the initial alignment line of the plate relative to the radius (Fig. 4c). The distal boundary of the plate position is set by the watershed line (Fig. 4d), marking the volar edge of the distal radius. A margin of 2mm proximal to this line is maintained, ensuring bony support but avoiding tendon irritation [7]. The watershed line is calculated by identifying, for each Y-slice, the mesh point with maximum X-value, then smoothing the curve with polynomial regression. Only points within 3 units of the mean Z-value are retained; this empirically chosen threshold best captured the anatomical contour without excessive flattening or irregularity.

**Fig. 5:**
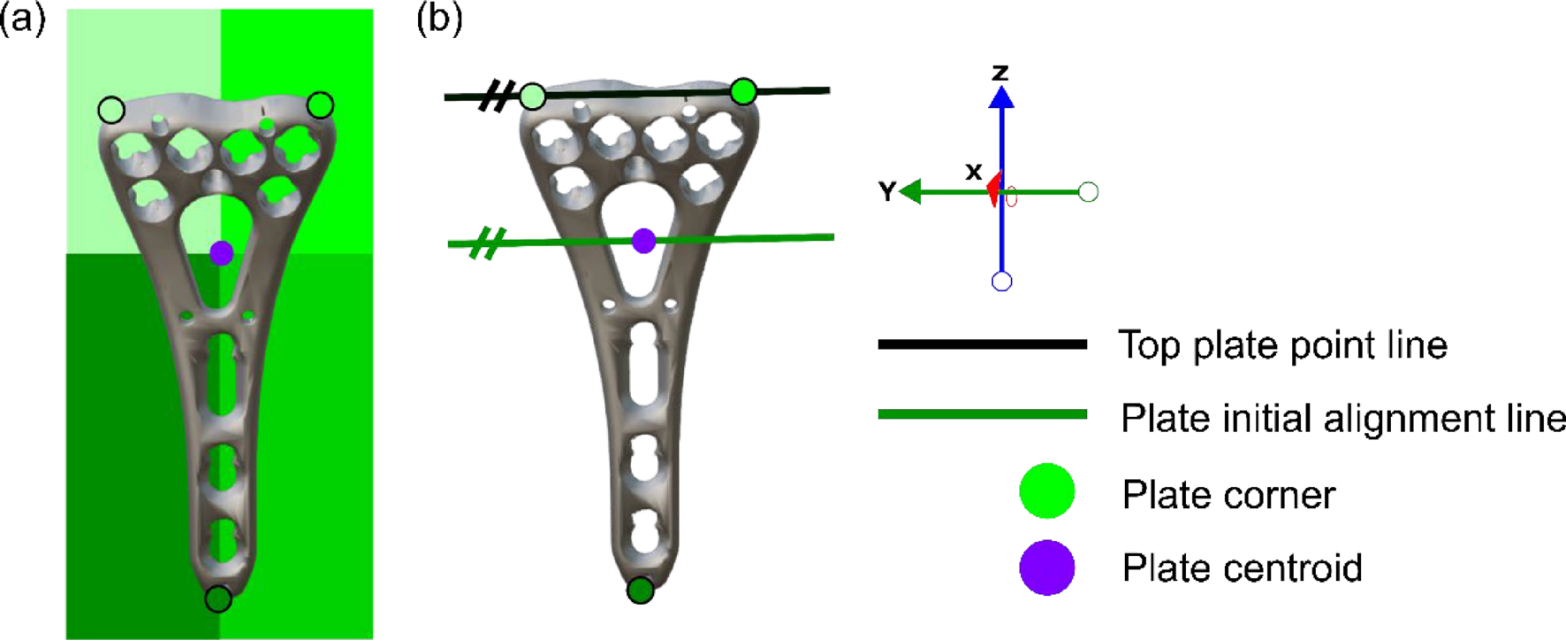
Algorithm step b in detail: Finding landmarks; plate. **a** Plate centroid (purple) and the quadrant corner points (green) are identified. **b** A line (black) connects the two top plate points. A parallel initial plate alignment line (green) is constructed through the centroid

To define plate landmarks, the plate is divided into four quadrants using PCA (Fig. 5a). The centroid is computed, and the vertices furthest from the centroid in the two upper quadrants define the top corners. A line is drawn connecting these top corners (Fig. 5b). The bottom corner is the vertex furthest from this line. Finally, the initial alignment line is constructed through the centroid, parallel to the top plate point line. This plate alignment line, combined with the bone initial alignment line, determines the initial plate position.

#### Algorithm Step (c): Initial Alignment

**Fig. 6:**
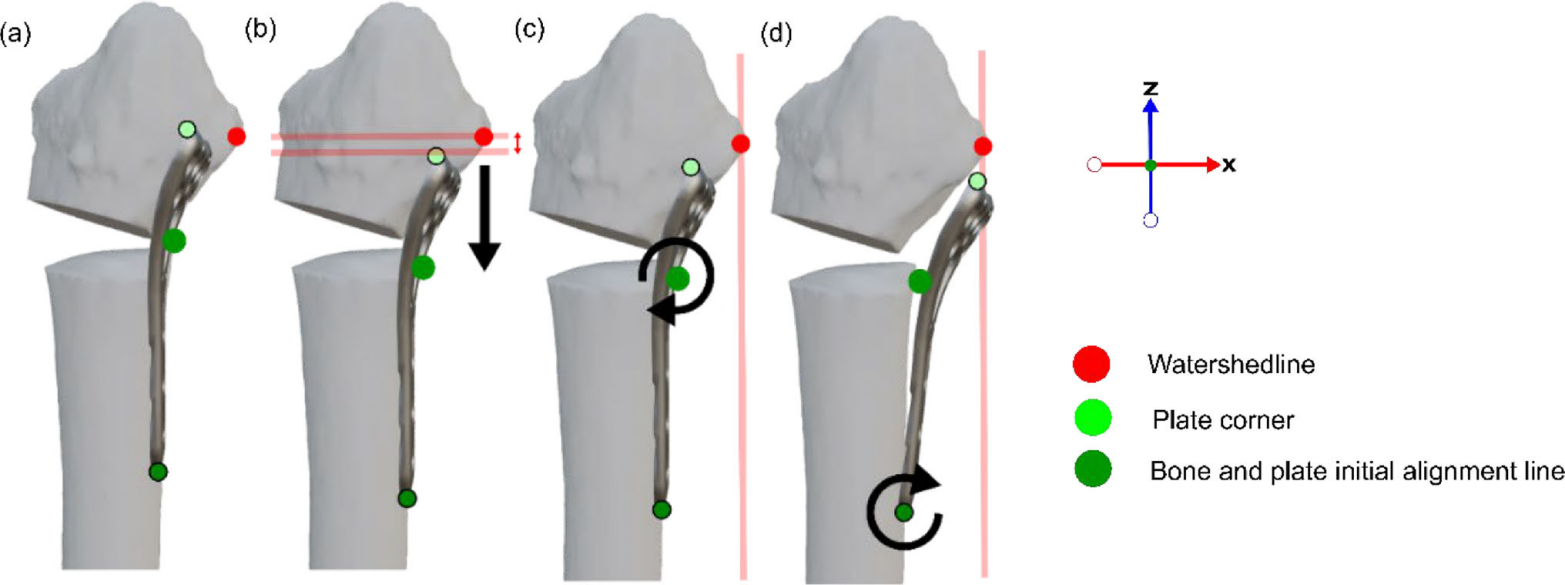
Algorithm step c in detail: initial alignment. **a** Bone and plate initial alignment lines (green) are overlapped. **b** Plate translated until its top edge lies ≥ 2mm below the watershed line (red). **c** Plate rotated so the bottom point contacts the radius surface. **d** Top plate corners aligned with the watershed line

A five-step alignment process positions the plate on the volar radius (Fig. 6):

First, the plate’s initial alignment line is overlapped with the radius’s initial alignment line (Fig. 6a). Second, plate translation along the Z-axis ensures its top corners lie at least 2 mm below the watershed line, (Fig. 6b). Third, the plate is rotated around its center line until the bottom point contacts the radius surface (Fig. 6c). Fourth, a second rotation aligns the plate's top corners with the watershed line X-values (Fig. 6d). Finally, the plate is moved toward the radius until at least one vertex intersects the bone surface.

#### Algorithm Step (d): Final Alignment

**Fig. 7:**
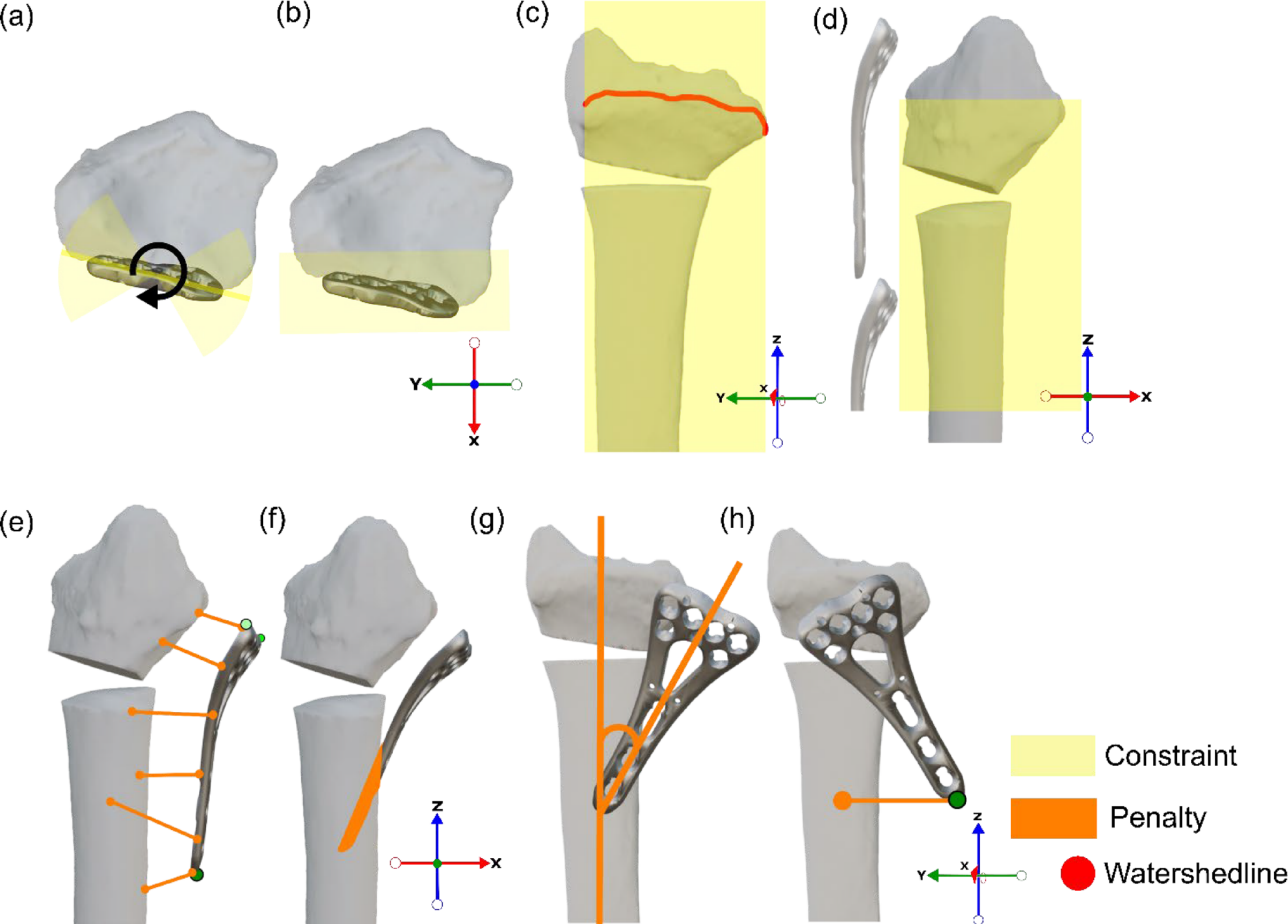
Algorithm step d in detail: final alignment, showing constraints (yellow) and penalties (orange). **a** Rotation constraint to ± 30 degrees from the initial alignment (XY-plane). **b** X-constraint: maximum ¼ radius. **c** Y-constraint: watershed line width. **d** Z-constraint: maximum 1.5 plate lengths from the bone mesh top. **e** Distance penalty, with extra weight for the three plate corners. **f** Inside bone mesh penalty. **g** Axis alignment penalty. H. Y-distance bottom vertex penalty

Plate orientation is optimized using the SLSQP algorithm [8] minimizing an objective function that balances penalties and constraints to ensure accurate placement without mesh penetration or extending beyond the volar radius (Fig. 7). In distal radius corrective osteotomies, the plate is placed flush with the bone surface within a defined range of rotation and translation. Constraints and penalties were defined based on clinical practice and expert consensus to cover all plausible positions while excluding anatomically or surgically unrealistic placements.

The minimizer is subjected to four spatial constraints (Fig. 7a–d): Rotations are limited to ± 30° around all three axes from the initial alignment, lateral displacement is limited to a quarter of the radius width, medial–lateral movement is restricted to the watershed line width, and vertical movement must remain within 1.5 plate lengths below the radius’s maximum Z-vertex without exceeding the watershed line’s Z-extent. To further refine placement, four penalties are applied (Fig. 7e–h): a distance penalty that minimizes the gap between plate and radius mesh points with increased weight for the three key corners, a mesh penetration penalty that prevents intrusion of plate vertices into the bone mesh, an axis alignment penalty that promotes directional coherence, and a Y-centering penalty that keeps the bottom plate point aligned with the shaft.

The objective function is defined as follows:

1 $$\begin{aligned}f\left(\mathrm{params}\right) &= {d}_{\mathrm{plate}} + a \cdot {d}_{\mathrm{top}}\\ &\quad + b \cdot {d}_{\mathrm{bottom}} + c \cdot {M}_{\mathrm{penalty}}\\ &\quad + d \cdot {\theta }_{\mathrm{penalty}} + e \cdot \left|{y}_{\mathrm{bottom}} - {y}_{\mathrm{middle}}\right| \end{aligned}$$

- The coefficients *a, b, c, d, e* represent adjustable weights corresponding to each penalty component, which were determined through an incremental trial-and-error approach, in which major constraints (distance between plate and bone) were prioritized over finer details (correct positioning of the proximal plate corner). Each weight was adjusted iteratively until the algorithm produced stable and clinically plausible plate placements across the dataset. For the radius, these weights were tuned to the following values: *a* = *105, b* = *100, c* = *4, d* = *5, e* = *60.*
- $d_{{{\mathrm{plate}}}}$are the distances from the transformed points to the bone vertices.
- $d_{{{\mathrm{top}}}}$ are the distances from the two top plate points to the bone vertices
- $d_{{{\mathrm{bottom}}}}$ is the distance from the bottom plate point to the bone vertices
- M_penalty_ is the number of plate vertices within the bone mesh
- $\theta_{{{\mathrm{penalty}}}}$ is the difference between the longest axes angles of plate and bone.
- y_bottom_ is the bottom plate point’s y-coordinate
- y_middle_ is the target bottom point y-coordinate on the middle of the radius.

### Algorithm Validation

Retrospectively, all patients were identified who underwent a 3D-planned corrective osteotomy of the distal radius at the Centre for Orthopaedic Surgery, OCON. Patients with a plate in situ, with plates that required intraoperative bending, or with different plate types were excluded, leaving 34 cases with a planned radius and corresponding plate position. For each patient, 3D preoperative planning had already been performed as part of the original clinical workflow, independent of this study. This planning included semi-automatic segmentation of the affected radius from preoperative CT scans using Mimics (version 24.0, Materialise NV, Leuven, Belgium), manual definition of the osteotomy plane, distal fragment realignment, and virtual placement of the fixation plate in its intended postoperative position.

### Time Comparison

Two internal experts, both experienced in weekly 3D preoperative surgical planning, provided an estimated manual planning time for plate positioning. This was supplemented by a prospective time trial in which a single expert manually positioned plates for a random subset of 10 patients from the study cohort while recording the time required (mean, SD, and 95% CI).

The runtime of the algorithm was measured for all 34 radii on two devices with different specifications: Device 1 (Intel i7-11800H, 2.30 GHz, 32 GB RAM, 64-bit) and Device 2 (Intel Pentium Gold 8505, 1.20 GHz, 4 GB RAM, 64-bit). On Device 1, additional analyses were performed to assess the effect of mesh density on runtime. Three datasets were created: a reference set with the original radius files, a decimated set reduced to one-sixth of the vertices (Decimated dataset), and a refined set with approximately four times the original number of vertices (Refined dataset). For all analyses, the same plate STL file was used (24,204 vertices).

### Comparison to Manual Plate Placement

The algorithm's final plate position was compared to manual placement using the mean unpaired maximum Hausdorff distance [9], total 3D rotation, and total 3D translation, reported with mean, SD, and 95% CI. Total plate rotation and translation represent the manual adjustment necessary after algorithm placement to reach the same position as the manual placement. The same analysis was applied to the Decimated and Refined datasets.

### Preliminary Further Application Testing

To assess robustness and applicability to different osteotomy–plate settings, the algorithm was additionally tested with three distal radius plates and a tibia–plate combination. For the radius plates, no changes to the algorithm were made to test direct compatibility. For the tibia, algorithm adaptations were implemented and documented.

## Results

### Time Comparison

Manual positioning time was estimated by two experts at approximately 30 min. In the prospective time trial, a single expert required 12.45 ± 4.56 min (*n* = 10, 95% CI 9.22–16.28 min).

**Fig. 8:**
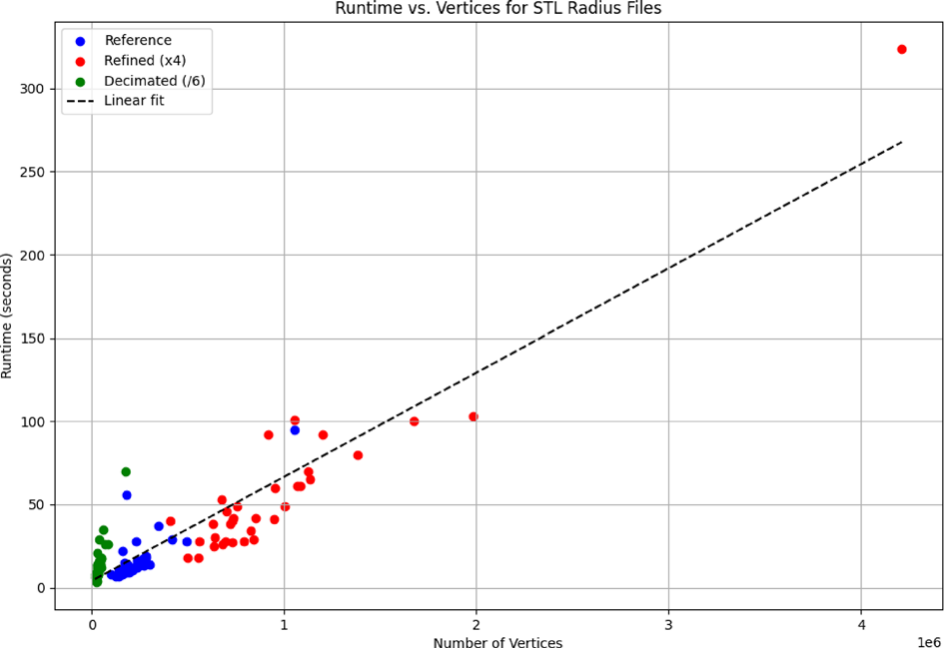
Correlation between number of vertices and runtime (seconds) from Reference, Refined and Decimated sets combined

The algorithm achieved a mean runtime of 18.3 ± 16.8 s on Device 1 (95% CI 12.4–24.1 s) and 27.7 ± 7.7 s on Device 2 (95% CI 25.0–30.4 s). On Device 1, runtimes for the Decimated and Refined datasets were 14.9 ± 12.1 s (95% CI 10.7–19.2 s) and 58.2 ± 53.1 s (95% CI 39.6–76.7 s), respectively. The correlation between runtime and vertex count was strong, as calculated across the Reference, Decimated, and Refined datasets, (*r* = 0.92, *R*2 = 0.85, *p* < 0.001; Fig. 8).

### Comparison to Manual Plate Placement

**Fig. 9:**
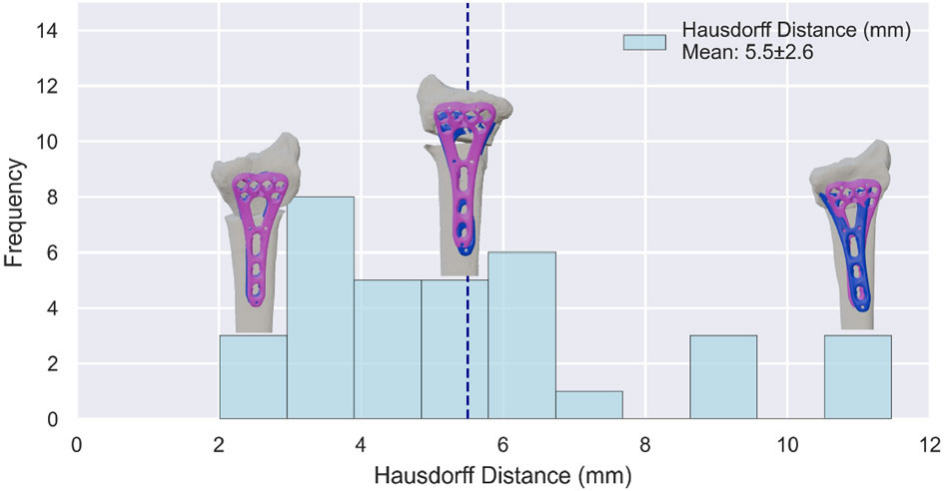
Histogram of maximum Hausdorff distances between the manual and algorithm plate placements

**Fig. 10:**
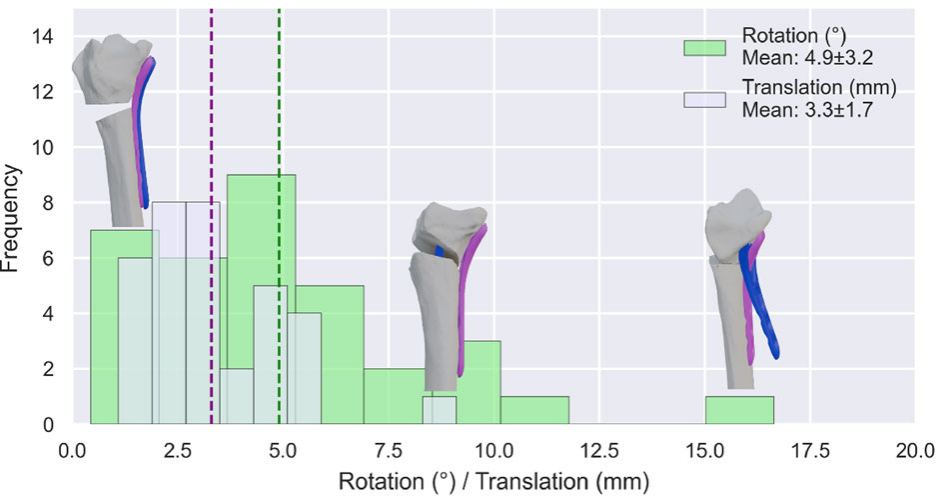
Histogram of total rotation and translation differences between the manual and algorithm plate placements

The results from the comparison to the manual placement, in terms of Hausdorff distance and rotation and translation, are presented in Figs. 9 and 10, respectively. The comparison for the three subsets is presented in Table 2.

**Table 2:** Results after comparison to manual positioning for the Reference, Refined, and Decimated set

Set	Hausdorff distance (mm)	Translation (mm)	Rotation (°)
Reference	5.5 ± 2.6 (95% CI 4.6–6.4)	3.3 ± 1.7 (95% CI 2.8–3.9)	4.9 ± 3.2 (95% CI 3.8–6.0)
Refined	5.5 ± 2.4 (95% CI 4.7–6.3)	3.6 ± 1.6 (95% CI 3.0–4.2)	4.2 ± 2.8 (95% CI 3.2–5.2)
Decimated	5.2 ± 2.4 (95% CI 4.4–6.0)	3.3 ± 1.6 (95% CI 2.7–3.9)	4.6 ± 3.6 (95% CI 3.4–5.9)

### Preliminary Further Application Testing

**Fig. 11:**
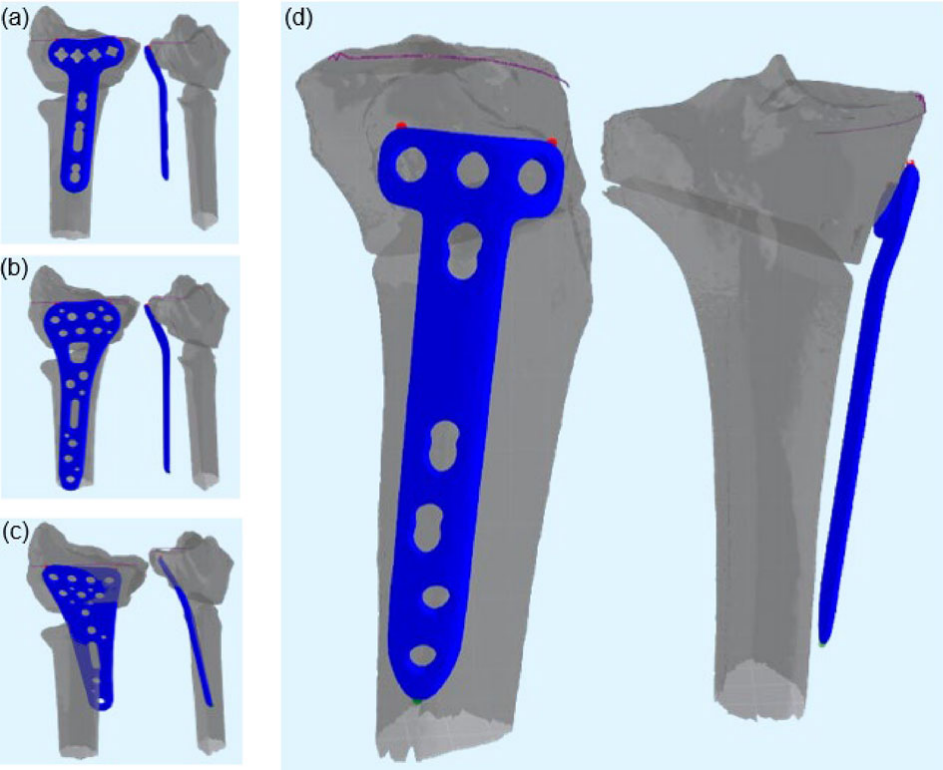
Results of preliminary further application testing for **a**, **b**, **c**: three different distal radius plates. **d**: A tibia with a tibial osteosynthesis plate

Figure 11 shows the results of applying the script to three distal radius plates (Fig. 11a–c) and to a tibia with a tibial plate (Fig. 11d). For the radius plates, the algorithm was used without any modifications. In the tibia case, only minor adjustments were required: The cutoff height was set to –100 to account for the larger bone size, the plate was rotated 180° around its longitudinal axis during initial alignment to face the correct side to the tibia, and the penalty weight for the bottom point was doubled to compensate for lower vertex density at the bottom corner.

## Discussion

This study presents an algorithm that automatically positions fixation plates in 18.3 ± 16.8 s, compared with 12.45 ± 4.56 min for manual placement. However, the measured manual time likely underestimates true clinical planning, since decisions such as plate choice and bending were predetermined. Evaluation of accuracy showed a mean unpaired maximum Hausdorff distance of 5.5 ± 2.5 mm (95% CI 4.6–6.4) (Fig. 9). The algorithm therefore provides a reliable starting position that substantially reduces manual effort, while still requiring minor adjustments in some cases to achieve the final clinically optimal placement. Runtime scaled approximately linearly with mesh density but remained under two minutes even in refined datasets, and decimation reduced runtime without compromising accuracy, indicating computational robustness across a range of inputs.

From a clinical perspective, the reported deviations (5.5 mm Hausdorff, 4.9° rotation, 3.3 mm translation) may appear large relative to typical corrections of a few millimeters or degrees. However, there is no single “correct” plate position: Multiple slightly different placements may be equally acceptable if the plate is flush with the bone, does not protrude, and provides stable fixation. This inherently limits the interpretation of deviations relative to a single expert reference, as it remains unknown whether the observed differences fall within or beyond the range of placements that other experts would consider clinically equivalent. Inter-expert variability has not yet been quantified for distal radius plate positioning. Therefore, our results should be viewed as quantifying reproducibility relative to one expert placement rather than defining absolute thresholds of clinical acceptability.

These findings support the use of the algorithm as an efficient generator of reliable initial placements that can be brought within clinical tolerance by small manual adjustments. Future validation efforts should include multiple independent expert placements to better capture the variability of acceptable solutions and define formal tolerance thresholds based on consensus across surgeons.

Compared with prior work, our contribution is complementary. Carillo et al. [10] and Carillo et al. [4] presented extensive pipelines in which minimizing plate-to-bone distances was only one steps. However, these studies did not report placement times or validate against manual plans. Our work focusses directly on efficiency and reproducibility, validated against manual placement. Methodologically, we deviated from ISB coordinate system guidelines [8] in favor of a computational approach that prioritizes simplicity and facilitates automation while ensuring consistent and reproducible orientation.

Several limitations must be acknowledged. The dataset was modest in size (*n* = 34), reflecting available clinical cases. While no a priori sample-size calculation was possible, effect sizes with 95% confidence intervals were reported to quantify precision, and consecutive case inclusion minimized selection bias. Errors such as plate–bone penetration (15/34 cases) and Y-axis deviations (6/34 cases) were observed, as previously described by Carrillo et al. [4]. While penalty adjustments can partially address these issues, the errors are also influenced by anatomical variation. Manual tuning of objective function weights was another limitation, as it is user-dependent and may not yield globally optimal values.

Future improvements should include replacing fixed thresholds (e.g., cutoff height of 40 mm, 17% of the average radius length, and smoothing factor of 3) with relative values to enhance generalizability across datasets, and formal evaluation of clinically acceptable placement constraints across larger cohorts. Automated parameter optimization, using manual plans as training data, could improve reproducibility by systematically adjusting penalty weights. Solver choice is also relevant: While the SLSQP solver from the SciPy library was effective at handling multiple constraints, as a gradient-based method it is sensitive to local minima, underscoring the need for accurate initial plate positioning [11]. Incorporating plate bending, which is common in practice, would further increase clinical realism.

In addition to these limitations, preliminary application to other plates and bones suggests that the framework is adaptable. Initial testing with different radius plates (Fig. 11a–c) shows that the core system functioned without modification, though tuning of penalty terms was required for each plate type. Application to the tibia with a tibial plate (Fig. 11d) produced a reasonable result after only minor adjustments. These findings indicate that the modular design is transferable in principle, but anatomy- and plate-specific refinements remain necessary before reliable use in broader clinical contexts.

In conclusion, this algorithm substantially reduces plate positioning time while providing reliable initial placements that require only minor manual refinement. Its modular design supports adaptation to other osteotomy–plate corrections, with the potential to improve efficiency and reproducibility in 3D surgical planning.

## Data Availability

The code and data will be available upon reasonable request.
